# Acoustic-driven self-rotating cylinders

**DOI:** 10.1038/s41598-026-51486-y

**Published:** 2026-05-06

**Authors:** Ali Ashrafian, Milad Tehranifar, Majid Rajabi

**Affiliations:** 1 Department of Mechanical Engineering, Sharif University of Technology, Tehran, Iran; 2 School of Mechanical Engineering, Sustainable Manufacturing Systems Research Laboratory, Iran University of Science and Technology, Tehran, Iran

**Keywords:** Acoustic-driven swimmers, Acoustic radiation torque, Acoustophoresis, Micro-robotics, Engineering, Mathematics and computing, Physics

## Abstract

This paper introduces the concept of ultrasound acoustic self-rotating cylinders, in which the source of self-rotation is due to the acoustic field radiation into the ambient fluidic environment. For a monochromatic radiating cylindrical body, we derive analytical expressions for the acoustic radiation torque and force and show that for particular designs of imposed normal velocity patterns over the cylinder’s surface, the wave-solid-fluid interactions lead to the exertion of non-zero torque on the body while maintaining zero net motion. The mathematical modeling of the self-excitation of the body is simplified as a distribution of normal velocities across the cylinder’s boundary with specified amplitude, frequency, and phases. We then propose several simple and versatile scenarios of velocity distribution and introduce several cases of desirable distributions as design strategies for self-rotating cylinders, which are validated numerically. Assuming a low Reynolds number condition, the frequency-dependent rotation velocity is estimated as a function of design parameters, and the feasible operating conditions are obtained.

##  Introduction

The attractive phenomena of acoustic radiation force^1^ and torque^2–4^ are known as the nonlinear effects caused respectively by the linear and angular momentum transfer from the acoustic field propagating in the host medium to an obstacle.

More than two decades have passed from the first proposals of utilizing these phenomena for noncontact handling, reorienting, and trapping of suspended micron- and milli-sized objects^5–7^.

Simultaneous with the development of optical manipulation of particles, the search for more sophisticated boundary conditions on the target object soared for applications such as the generation of negative radiation force and precision particle manipulation^6,8,9^. Researchers have consequently tried to induce phase and/or amplitude asymmetries in the incident wave in order to achieve the intended boundary conditions. Changing the focus from the incident to the scattered and radiated waves, the boundary of the target objects or their surroundings has been subject to vibration, heating, and topological modifications that allow control of the scattered field and tailoring the resulting acoustic radiation force and torque. For example, the repulsion, attraction, and cloaking effects associated with a radiating cylindrical body in the vicinity of half-space and corner space boundaries were discovered in^10^. Winckelmann and Bruus derived analytical results for the effect of temperature gradients on the speed of sound and, subsequently, the radiation force experienced by submerged microparticles^11^.

The efficiency of acoustic manipulation was recently enhanced by introducing the concept of the acoustic active (i.e., radiating) bodies with advanced applications as carriers or drug, bioactive agent, and material delivery assets^12–15^. A counter-intuitive feature due to the radiation effect is unveiled as the emergence of non-zero acoustic radiation force associated with the partially acoustic radiation of a body in the absence of an incident wave field^15^. The mechanism designed for transferring linear momentum flux is offered by the authors as the self-propulsive driver.

Likewise, the transfer of acoustic angular momentum has been studied theoretically and experimentally^16,17^. Fan et al. used a 64-element annular piezoelectric ceramic array immersed in a water-filled chamber to impose a linear azimuthal phase ramp; the resulting first-order acoustic vortices produced clear rotational streaming patterns with phase maps reproducing the imposed sectoral excitation^18^. Hong et al. generated the same helical wavefronts in air using three to six peripherally placed transducers; the orbital angular momentum carried by these vortex fields spun water droplets at rates exceeding hundreds of revolutions per second^19^. Similarly, Toninelli et al. constructed a compact free-space “acoustic spanner” from eight miniature loudspeakers driven with phased signals and a 3D-printed funnel concentrator; the helical sound beam rotated macroscopic objects (e.g. 3 g polystyrene packing peanuts) by reversing the topological charge. In every case the individual piezoelectric sectors or speaker cones vibrated locally, yet the overall structure allowed independent phase control sufficient to realize a clean sectoral velocity distribution and transfer measurable torque^20^. In another closely related context, VolkeSepúlveda et al. demonstrated the transfer of orbital angular momentum from acoustical vortex beams to suspended disks in free space, where the torque arises from the interaction between the object and an externally imposed helical wave field^21^.

Following recent studies on acoustically driven swimmers with internal excitation mechanisms^22,23^, the feasibility study of a self-rotating system composed of a self-radiating cylindrical body is presented in this paper in order to investigate the possibility of non-zero torque generation on the cylindrical body, as a model for acoustic self-activated rotors. Several stimulation patterns are studied, and the resultant radiation force and torque are compared as a function of frequency and pattern design. Notably, the interesting cases of vanishing force and non-zero radiation torque are discussed and it is shown that for any centrally symmetric or antisymmetric pattern of phase excitation of the rotor, such non-zero net torques will be produced. Assuming a low Reynolds number condition, the frequency-dependent rotation velocity is estimated as a function of design parameters, and the performance of the device is discussed.

Considering the practical limitations, the stimulation patterns are proposed according to a multiple-partial-sections design scheme so that the partial sections are assumed to be equipped with independent but monochromatic time harmonic excitation internal drivers. In practice, the sector-phased normal-velocity boundary can be realized by wrapping and bonding ultrathin piezoelectric rings onto rigid metallic cylinders (e.g., stainless steel). Flexible phased arrays based on embossed polymer piezo films and thin-film PZT transferred to polyimide have demonstrated conformal mounting and electronic phase control at small radii, while sol-gel PZT flexible ultrasonic transducers have been bonded to steel pipes and operated at elevated temperature, directly supporting our ring-on-steel self-rotary cylinder. Related annular/circular arrays have also been used to shape fields and drive streaming in confined fluids (e.g., annular arrays generating controllable acoustic-vortex patterns), underscoring the practicality of sector-addressed excitation; meanwhile, MEMS-scale thin-film PMUT platforms and flexible array demonstrations document mature routes for dense, addressable element patterning^24–27^..

##  Mathematical formulations

### Problem definition

**Fig. 1:**
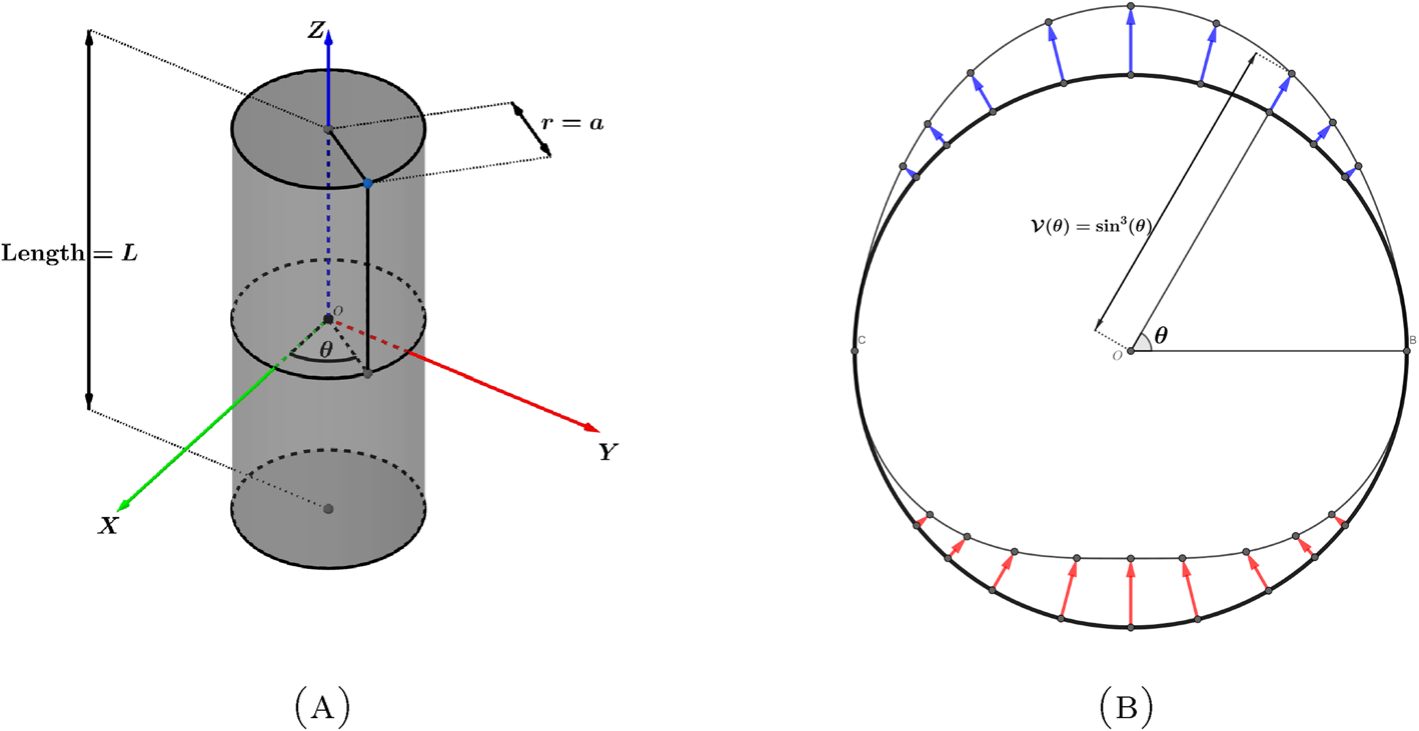
(a) The geometry of the self-rotating cylinder with radius *a* and length *L*. (b) The lateral boundary of the cylinder is vibrating according to $\boldsymbol{v}(a,\theta )=\mathcal {V}(\theta )=\sin ^3(\theta )$.

Let $\Omega$ be a circular cylinder of radius $r=a$ and length *L* submerged in an unbounded fluid whose density, pressure and velocity fields are denoted by $\rho _t(\boldsymbol{x},t)$, $p_t(\boldsymbol{x},t)$ and $\boldsymbol{v}_t(\boldsymbol{x},t)$, respectively. The subscript t stands for the total (ambient and perturbed) acoustic fields. Assuming the acoustic wavelength to be much larger than the thermal and viscous boundary layers, the fluid can be modeled as inviscid, which simplifies the governing Navier-Stokes equations into Euler equations^28^. A cylindrical coordinate system $\boldsymbol{x}=(r,\theta ,z)$, positioned at the center of the cylinder, is introduced (See Fig. 1a). The lateral boundary of the cylinder, $\partial \Omega$, is assumed to vibrate according to an a priori known function $\boldsymbol{v}_t(a,\theta )=\mathcal {V}(\theta )$, as shown in Fig. 1b. The cylinder is taken to be sufficiently long ($L>>a$) so that the end effects become negligible, allowing the problem to reduce to a two-dimensional one. General families of $\mathcal {V}(\theta )$ are sought that produce self-rotation of the cylinder; i.e. the rotation of the cylinder about its axis while the cylinder experiences no net motion.

###  Wave Dynamics

The fields introduced in Section Problem definitionare interrelated via the conservation of mass and momentum equations^29^:

1a $$\begin{aligned} \frac{\partial \rho _t}{\partial t}+\nabla \cdot (\rho _t\boldsymbol{v}_t)&=0\end{aligned}$$

1b $$\begin{aligned} \rho _t\frac{\partial \boldsymbol{v}_t}{\partial t}+\rho _t(\boldsymbol{v}_t\cdot \nabla )\boldsymbol{v}_t+\nabla p_t&=\boldsymbol{0} \end{aligned}$$

Small perturbations are assumed around mean values ($p_t-p_0=\epsilon p_1=\tilde{p}$, $\rho _t-\rho _0=\epsilon \rho _1=\tilde{\rho }$, and $\boldsymbol{v}_t=\epsilon \boldsymbol{v}_1=\tilde{\boldsymbol{v}}$), where $\tilde{p}, \tilde{\boldsymbol{v}},$ and $\tilde{\rho }$ are the perturbed acoustic fields and $\epsilon =\frac{V_0}{c_0}$ is the perturbation factor defined using the characteristic velocity $V_0$. Using the perturbed fields, Eq. 1a and 1bcan be linearized as^29^:

2a $$\begin{aligned} \frac{\partial \tilde{\rho }}{\partial t}+\rho _0\nabla \cdot \tilde{\boldsymbol{v}}&=0\end{aligned}$$

2b $$\begin{aligned} \frac{\partial \tilde{\boldsymbol{v}}}{\partial t}+\frac{1}{\rho _0}\nabla \tilde{p}&=\boldsymbol{0} \end{aligned}$$

where the thermodynamic state variable $\tilde{p}$ depends on the entropy of the system, *s*, and its density $\tilde{\rho }$. From now on, the tilde symbol of the fluctuation terms will be dropped for brevity. According to Laplace’s hypothesis, we assume the entropy of fluid particles to remain constant along their trajectory:

3 $$\begin{aligned} \frac{Ds}{Dt}=0\quad \frac{D}{Dt}:=\frac{\partial }{\partial t}+\boldsymbol{v}\cdot \nabla \end{aligned}$$

We further take the fluid to be initially isentropic. Eq. 3 then suggests that the medium will retain its isentropic characteristic for all times, based on which we approximate the pressure field as a linear function of density using the Taylor expansion of equation:

4 $$\begin{aligned} p(\rho )=p(\rho =0)+\left( \frac{\partial p}{\partial \rho }\right) _{s}\rho +\mathcal {O}(\rho ^2)=c_0^2\rho \end{aligned}$$

where $c_0=\sqrt{\left( \frac{\partial p}{\partial \rho }\right) _{s}}$ is the adiabatic speed of sound. Note that the higher order terms in Eq. 4 have been discarded to be consistent with the first order perturbation assumption. Moreover, Eq. 2b indicates that the fluid is irrotational, allowing us to write the velocity and subsequently the pressure fields in terms of a scalar potential $\phi (\boldsymbol{x},t)$ such that

5a $$\begin{aligned} \boldsymbol{v}&=-\nabla \phi \end{aligned}$$

5b $$\begin{aligned} p&=\rho _0\frac{\partial \phi }{\partial t} \end{aligned}$$

Plugging Eq. 5 and 3 into Eq. 2 and after some algebraic manipulations, we derive the wave Eq. 6 governing the motion of fluid particles:

6 $$\begin{aligned} \frac{1}{c_0^2}\frac{\partial ^2\phi }{\partial t^2}-\nabla ^2\phi =0 \end{aligned}$$

For monochromatic waves, all acoustic field variables have $e^{-i\omega t}$ as their time-dependence factor corresponding to the constant wave frequency $\omega$ and wavenumber $k=\frac{\omega }{c_0}$ in the frequency domain. We can therefore take the Fourier transform of Eq. 6 to arrive at

7 $$\begin{aligned} \nabla ^2 \psi (\boldsymbol{x},\omega )+k^2\psi (\boldsymbol{x},\omega ) = 0 \end{aligned}$$

where $\psi (\boldsymbol{x},\omega )=\int _{-\infty }^{\infty }\phi (\boldsymbol{x},t)e^{i\omega t}\textrm{dt}$ is the scalar potential in frequency domain and $\nabla ^2$ denotes the scalar Laplacian operator. In the next sections, we will solve this equation for a family of boundary conditions imposed upon $\partial \Omega$. Once $\psi$ is obtained in terms of $\mathcal {V}(\theta )$, the radiation tensor will be calculated to find the acoustic radiation force and torque experienced by the cylinder.

###  Derivation of field variables

For sufficiently long cylinders, the solution to Eq. 7 can be written in the following form^30^:

8 $$\begin{aligned} \psi (r,\theta ,\omega )=\psi _0\sum _{n=0}^{\infty }(A_n\sin (n\theta )+B_n\cos (n\theta ))H_n^{(1)}(kr) \end{aligned}$$

where $H_n^{(1)}(kr)$ is the cylindrical Hankel function of the first kind^31^, $\psi _0$ is the amplitude of the velocity potential, and $A_n$ and $B_n$ are the scaled scattering coefficients to be calculated after imposing boundary conditions. Note that the Sommerfeld radiation condition has been already applied to eliminate incoming waves at infinity.

**Fig. 2:**
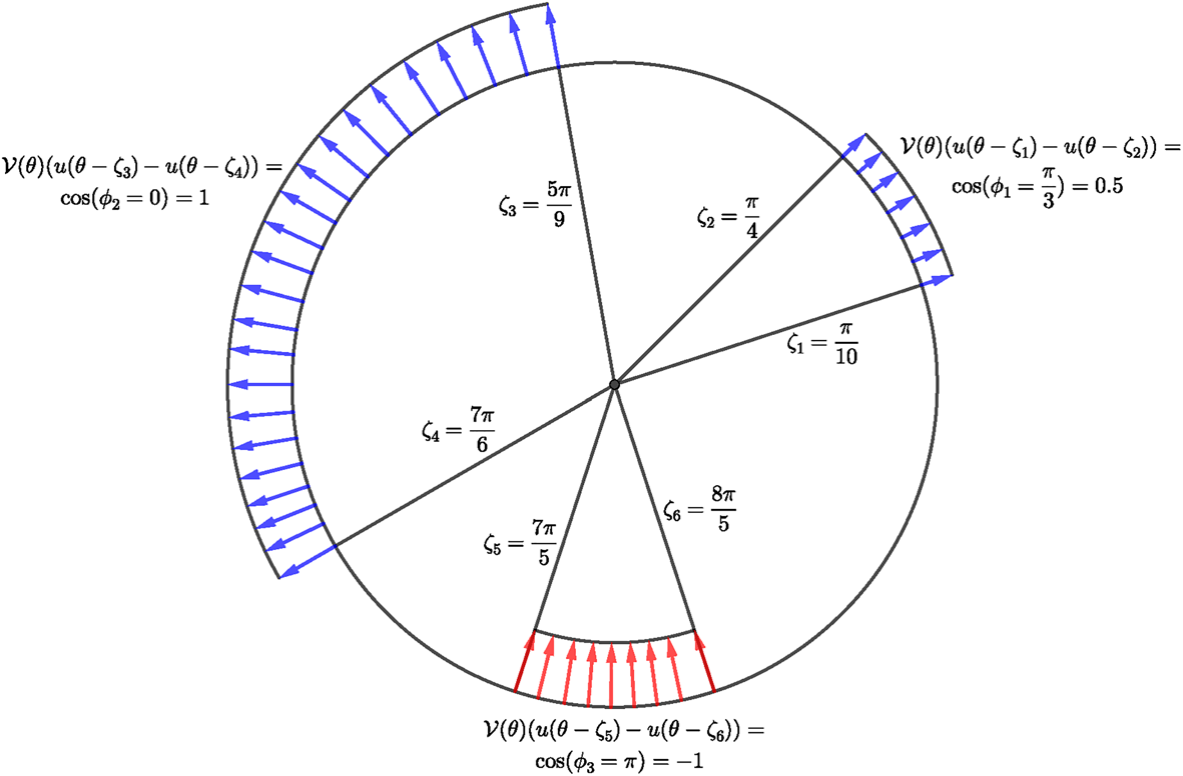
Schematic of a boundary velocity profile at t=0 with three sectors $(\zeta _1=\frac{\pi }{10},\zeta _2=\frac{\pi }{4})$, $(\zeta _3=\frac{5\pi }{9},\zeta _4=\frac{7\pi }{6})$ and $(\zeta _5=\frac{7\pi }{5},\zeta _6=\frac{8\pi }{5})$ which have phases $\phi _1=\frac{\pi }{3}$, $\phi _2=0$ and $\phi _3=\pi$ respectively. (The blue/red color is associated with outward/inward direction of vibration.).

The scattering coefficients can be uniquely determined by imposing a Neumann boundary condition, which is equivalent to matching the normal velocity of the fluid at $r=a$ with that of the surface of the cylinder. The focus of this paper is on velocity profiles whose phase distribution varies linearly across the boundary while a constant vibration amplitude is preserved in the time domain. Specifically, a set of N ordered pairs $(\zeta _{2i-1},\zeta _{2i})$ are used to configure N arbitrary, non-overlapping circular sectors of the cylinder’s cross-section, such that the $i^{\text {th}}$ sector starts at angle $\zeta _{2i-1}$ and ends at angle $\zeta _{2i}$ (Fig. 2). Furthermore, the cylinder’s boundary in the $i^{th}$ sector is assigned a velocity of constant amplitude $V_0$ and a phase equal to $\phi _i$. Mathematically:

9 $$\begin{aligned} \boldsymbol{v}^*(\theta ,t)=\mathcal {V}(\theta )e^{-\textrm{i}\omega t}=V_0\sum _{k=1}^N\left( u(\theta -\zeta _{2k-1})-u(\theta -\zeta _{2k})\right) e^{\textrm{i}\phi _k}e^{-\textrm{i}\omega t} \end{aligned}$$

where $\boldsymbol{v}^*$ is the displacement field on $\partial \Omega$ and $u(\theta )$ is the unit step function. Considering the relation $\boldsymbol{v}=e^{-\textrm{i}\omega t}\nabla \psi$, we impose the following boundary condition on $\partial \Omega$:

10 $$\begin{aligned} \nabla \psi .\boldsymbol{n}\Bigr |_{\begin{array}{c} r=a \end{array}}=\frac{\partial \psi }{\partial r}\Bigr |_{\begin{array}{c} r=a \end{array}}=V_0\sum _{k=1}^Ne^{\textrm{i}\phi _k}\left[ u(\theta -\zeta _{2k-1})-u(\theta -\zeta _{2k})\right] \end{aligned}$$

It should be clarified that the boundary condition in Eq. (10) implicitly assumes a rigid cylinder. If the cylinder were fully elastic, local vibration of one sector would induce global structural waves, leading to mechanical crosstalk and making it problematic to impose the exact piecewise velocity distribution $\mathcal {V}(\theta )$. However, following the classic analysis of Hasegawa (1969)^32^ for elastic scatterers, the deviation from rigid behaviour becomes significant only near the natural frequencies of the cylinder. Far from resonance, the rigidity assumption is a well-established approximation used to derive closed-form equations for radiation force and torque in the literature^1,33,34^.

In Eq. 10, $\boldsymbol{n}$ denotes the outward normal vector of $\partial \Omega$. Substituting Eq. 8 into Eq. 10, it can be inferred that the angular component of the velocity field in Eq. 8 is actually the Fourier series expansion for $\mathcal {V}(\theta )$, allowing us to obtain the scattering coefficients in terms of the phase distribution and sectors’ configuration. Using the orthogonality of the trigonometric system, we have:

11 $$\begin{aligned} k\psi _0{H_n^{(1)}}^{'}(ka) \begin{Bmatrix} B_n\\ A_n\\ \end{Bmatrix} =\frac{V_0\epsilon _n}{2\pi }\left( \sum _{m=1}^N\int _{\zeta _{2m-1}}^{\zeta _{2m}}e^{\textrm{i}\phi _m} \begin{Bmatrix} \cos (n\theta )\\ \sin (n\theta )\\ \end{Bmatrix} \textrm{d}\theta \right) \quad (n\ge 0) \end{aligned}$$

where $\epsilon _n = 1+u(n)$ is the Neumann symbol^35^. The integrals in Eq. 11 can be further simplified to Eq. 12, considering the constancy of $\phi _m$ in the interval $(\zeta _{2m-1},\zeta _{2m})$.

12a $$\begin{aligned} \begin{Bmatrix} B_n\\ A_n\\ \end{Bmatrix} =&\frac{V_0}{n\pi k\psi _0{H_n^{(1)}}^{'}(ka)}\sum _{m=1}^Ne^{\textrm{i}\phi _m} \begin{Bmatrix} \sin (n\zeta _{2m})-\sin (n\zeta _{2m-1}) \\ \cos (n\zeta _{2m-1})-\cos (n\zeta _{2m})\\ \end{Bmatrix} \quad (n\ge 1)\end{aligned}$$

12b $$\begin{aligned} \begin{Bmatrix} B_0\\ A_0\\ \end{Bmatrix} =&\frac{V_0}{2\pi k\psi _0{H_0^{(1)}}^{'}(ka)} \begin{Bmatrix} \sum _{m=1}^Ne^{\textrm{i}\phi _m}(\zeta _{2m}-\zeta _{2m-1})\\ 0\\ \end{Bmatrix} \end{aligned}$$

Equations 12a and 12b directly relate the scattering coefficients $A_n$ and $B_n$ to the geometrical and phase characteristics of a given boundary velocity. This will pave the way for establishing a direct relation between acoustic radiation torque and force and the input design parameters.

###  Acoustic radiation force and torque

####  Acoustic radiation force (ARF)

In an inviscid fluid, the fluid parcels at the interface with the cylinder vibrate with the same normal velocity as $\partial \Omega$. This propagates a harmonic perturbation into the fluid - governed by Eq. 7- which, itself, prompts further momentum transfer to the cylinder, often described using the momentum flux density tensor. For an inviscid fluid, this tensor is defined as^36^:

13 $$\begin{aligned} \Pi =p_t\mathbb {I}+\rho \boldsymbol{v}_t\otimes \boldsymbol{v}_t \end{aligned}$$

where $\mathbb {I}$ is the identity tensor and $\boldsymbol{a}\otimes \boldsymbol{b}$ denotes the outer product of vectors $\boldsymbol{a}$ and $\boldsymbol{b}$. The force exerted on the cylinder should be calculated by integrating the normal component of $\Pi$ over the cylinder’s surface. Notably, the oscillatory nature of the acoustic momentum flux tensor necessitates the use of the time-averaged stress tensor - also known as radiation or Brillouin tensor^37^- to calculate a corresponding time-averaged radiation force. Mathematically:

14 $$\begin{aligned} \langle \boldsymbol{F}\rangle =\oint \langle \Pi \rangle .\boldsymbol{n}\textrm{dA} \end{aligned}$$

with $\langle \boldsymbol{\cdot }\rangle =\frac{\omega }{2\pi }\int _{0}^{T}\boldsymbol{\cdot }\textrm{d}t$ being the operator that averages oscillating variables over one period. The averaged force in Eq. 14 is called the acoustic radiation force (ARF). It is well-known that ARF is a second-order effect^38^; i.e. the first-order perturbations of acoustic fields do not induce a force upon the target object. Nevertheless, the second-order radiation tensor can be rewritten in terms of the first-order quantities:

15 $$\begin{aligned} \langle \Pi \rangle =\left( \frac{\langle p^2\rangle }{2\rho _0c_0^2}-\frac{\rho _0\langle |\boldsymbol{v}|^2\rangle }{2}\right) \mathbb {I} + \rho _0\langle \boldsymbol{v}\otimes \boldsymbol{v}\rangle \end{aligned}$$

Taking time-average of the Eulerian conservation of momentum equation, we arrive at

16 $$\begin{aligned} \langle \frac{\partial (\rho \boldsymbol{v})}{\partial t} + \nabla .\Pi \rangle =\nabla .\langle \Pi \rangle =\boldsymbol{0} \end{aligned}$$

The solenoidal nature of the Brillouin tensor means that an imaginary surface at the far field such as $\partial \Omega _{\infty }$ can be used to calculate the ARF as a consequence of the divergence theorem^31^. By further writing the first order pressure and velocity fields in Eq. 15 in terms of $\psi$ and considering Eq. 15 and 8, the ARF (as well as the acoustic radiation torque or ART) are computed in terms of the scattering coefficients in Eq. 17 and 19, respectively.

17 $$\begin{aligned} \begin{aligned} \langle \boldsymbol{F}\rangle&=-\oiint _{\partial \Omega }\langle \Pi \rangle .\boldsymbol{n}\textrm{dA}=\lim _{kR \rightarrow \infty }\frac{1}{2}\rho k^2\oiint _{\partial \Omega _{\infty }}|\psi |^2\boldsymbol{e_r}\textrm{dA}\\&=-\rho _0{\psi _0}^2kL\sum _{n,m=0}^{\infty }\text {Re}\left\{ \textrm{i}^{m-n}\left( \gamma _{n,m}(A_nA_m^*+B_nB_m^*)\boldsymbol{i}+\lambda _{n,m}(A_nB_m^*-B_nA_m^*)\boldsymbol{j}\right) \right\} \\&=\frac{4\rho _0LV_0^2}{\pi ^2k}\sum _{n=0}^{\infty }\sum _{\begin{array}{c} j=1\\ k=1 \end{array}}^{N}\text {Im}\left\{ \frac{e^{\textrm{i}(\phi _j-\phi _k)}}{n(n+1){H_{n}^{(1)}}^{'}(ka){H_{n+1}^{(2)}}^{'}(ka)}\right\} \boldsymbol{\Upsilon }_{n,j,k} \end{aligned} \end{aligned}$$

where $\gamma _{n,m}=(2-\frac{\epsilon _n}{2})(2-\frac{\epsilon _m}{2})(\delta _{n,m-1}+\delta _{n,m+1})$ and $\lambda _{n,m}=(2-\frac{\epsilon _m}{2})(\delta _{m,n-1}-\delta _{n,m-1})$ are functios of the Neumann symbol ($\epsilon _n$) and the Kronecker delta ($\delta _{n,m}$). The vector coefficients $\boldsymbol{\Upsilon }_{n,j,k}$ are solely dependent on the configuration of the sectors and are consequently a function of $\zeta _i$’s (See Supplementary Eq. S1 to S15 for the full derivation). The Hankel functions that appear in the velocity potential are replaced by their asymptotic forms at the far-field. According to Eq. 17, the ARF is a linear combination of sinusoidal phase differences between any two sectors. It can subsequently be inferred that for each two sectors such as $(\zeta _{2j-1},\zeta _{2j})$ and $(\zeta _{2k-1},\zeta _{2k})$, their contribution to the resultant radiation force vanishes if either $\phi _j=\phi _k$ or $\phi _j=\phi _k\pm \pi$. However, imposing this boundary condition on all sectors will also prevent the cylinder from rotating. Taking a closer look at Eq. 17, the coefficients $\gamma _{n,m}$ and $\lambda _{n,m}$ dismiss all but the products of two consecutive scattering coefficients. Even and odd boundary velocity functions $\mathcal {V}(\theta )$ are therefore another option to make the radiation force vanish since they only have nonzero Fourier coefficients of a single parity (i.e. either $A_{2n}$ or $A_{2n-1}$ and either $B_{2n}$ or $B_{2n-1}$ equal zero for all integer values of *n*). Further in the paper, families of even and odd boundary conditions are investigated and their corresponding ART are derived.

####  Acoustic radiation torque

Similar to 2.4, the radiation torque is obtainable by integrating the infinitesimal torque vector $\textrm{d}{\boldsymbol{T}}=\boldsymbol{r}\times \boldsymbol{F}=\boldsymbol{r}\times (\Pi \cdot \boldsymbol{n})$ over $\partial \Omega$. It can be easily shown that

18 $$\begin{aligned} \boldsymbol{r}\times (\nabla \cdot \Pi )=\nabla \cdot (\boldsymbol{r}\times \Pi ) \end{aligned}$$

where the angular momentum flux density tensor is defined as $\boldsymbol{r}\times \Pi = \varepsilon _{ijk}x_j\Pi _{kl}\boldsymbol{e}_i\otimes \boldsymbol{e}_l$, with $\varepsilon _{ijk}$ being the Levi-Civita symbol^31^. The identity provided in Eq. 18 paves the way for employing far-field integration similar to the derivation of ARF in Eq. 17 (See Supplementary Eq. S16 to S25 for the full derivation):

19 $$\begin{aligned} \begin{aligned}&\langle \boldsymbol{T}\rangle \cdot \boldsymbol{k}=\langle T_z\rangle =-\oiint _{\partial \Omega }\boldsymbol{r}\times \Pi \cdot \boldsymbol{n}\textrm{dA}=-\lim _{kR \rightarrow \infty }\frac{1}{2}\rho _0\oiint _{\partial \Omega _{\infty }}\text {Re}\left\{ \frac{\partial \overline{\psi }}{\partial r}\frac{\partial \psi }{\partial \theta }\right\} \textrm{dA}\\&=2\rho _0L\psi _0^2\sum _{n=1}^{\infty }n\text {Im}\left\{ A_n^*B_n\right\} \mathop {=}\limits ^{\text {Eq. 11}}\frac{8\rho _0LV_0^2}{\pi ^2k^2}\sum _{n=1}^{\infty }\sum _{1\le i\le j}^{N}\frac{\sin (\phi _i-\phi _j)}{n|{H_n^{(1)}}^{'}(ka)|^2}\Lambda _{n,i,j}\\ \end{aligned} \end{aligned}$$

In Eq. 19, $\Lambda _{n,i,j}=\sin (n(\zeta _j^*-\zeta _i^*))\sin (n\frac{\Delta \zeta _j}{2})\sin (n\frac{\Delta \zeta _i}{2})$ is a geometry-dependent factor in which $\zeta _i^*=\frac{\zeta _{2i-1}+\zeta _{2i}}{2}$ and $\Delta \zeta _i=\zeta _{2i}-\zeta _{2i-1}$ denote the median point and the arc angle of the $i^{th}$ sector. Similar to Eq. 17, each mode of the radiation torque derived in Eq. 19 is a multiplication of phase and geometry-dependent factors. Eq. 19 is the general form of the ART for a self-radiating cylinder with an arbitrary number of sectors each vibrating with their pre-defined phase. The factor $\sin (\phi _i-\phi _j)$ in Eq. 19 reveals that geometrical asymmetries alone cannot induce a net torque upon the cylinder unless there exists a phase difference modulo $\pi$ between two or more sectors. We, therefore, restrict our attention to linearly varying phases in the rest of the paper.

####  Linearly varying phase distribution

As mentioned before, the main task of this paper is to find boundary conditions such as $\mathcal {V}(\theta )$ that allow the cylinder to rotate about its axis, without experiencing a net radiation force. For practical purposes, we limit our study to two family of boundary conditions with linearly varying phase which we call them the *central symmetric* and the *central anti-symmetric* phase distributions (see Eq. 20).

20 $$\begin{aligned} \begin{aligned}&\text {Central Symmetric (CS): }\phi _i=\frac{\phi _0\text {mod}(i-1,N_h)}{N_h-1} \\&\text {Central Anti-symmetric (CAS): }\phi _i=\frac{\phi _0\text {mod}(i-1,N_h)}{N_h-1} + \lfloor \frac{i-1}{N_h}\rfloor \pi \end{aligned} \end{aligned}$$

where $\text {mod}(a,n)$ denotes the remainder of *a* modulo *n*, $\phi _0$ is the maximum phase of the distribution which we call the “terminal phase”, and $N_h$ is the number of sectors in each half of the radiating cylinder. The cylinder is partitioned into $N=2N_{h}$ equiangular sectors to each of which a vibration of constant phase and amplitude in the frequency domain is assigned.

In the special case when equi-angular sectors partition the cylinder ($\zeta _{2i-1}=\frac{(i-1)\pi }{N_h}$, $\zeta _{2i}=\frac{(i)\pi }{N_h}$), Eq. 19 can be substantially simplified using the Lagrange’s trigonometric identity^39^. Plugging Eq. 20 into Eq. 19 and after algebraic manipulations, we arrive at

21a $$\begin{aligned}&<T_Z>_{\text {CS}}=\frac{-32\rho _0L\mathcal {V}_0^2}{k^2\pi ^2}\sum _{n=2,4,\cdots }^{\infty }\frac{\sin ^2(\frac{N_h\phi _0}{2(N_h-1)})\sin \left( \frac{\phi _0}{N_h-1}\right) \sin \left( \frac{n\pi }{N_h}\right) \sin ^2\left( \frac{n\pi }{2N_h}\right) }{\left[ \cos \left( \frac{n\pi }{N_h}\right) -\cos \left( \frac{\phi _0}{N_h-1}\right) \right] ^2n|{H_n^{(1)}}^{'}(ka)|^2}\end{aligned}$$

21b $$\begin{aligned}&<T_Z>_{\text {CAS}}=\frac{-32\rho _0L\mathcal {V}_0^2}{k^2\pi ^2}\sum _{n=1,3,\cdots }^{\infty }\frac{\cos ^2(\frac{N_h\phi _0}{2(N_h-1)})\sin \left( \frac{\phi _0}{N_h-1}\right) \sin \left( \frac{n\pi }{N_h}\right) \sin ^2\left( \frac{n\pi }{2N_h}\right) }{\left[ \cos \left( \frac{n\pi }{N_h}\right) -\cos \left( \frac{\phi _0}{N_h-1}\right) \right] ^2n|{H_n^{(1)}}^{'}(ka)|^2} \end{aligned}$$

where the tedious summations over each pair of sectors in Eq. 19 are relieved, making Eq. 21 interpretable in terms of the input parameters $\phi _0$, $N_h$, and *ka*. These parameters completely define the dimensionless radiation torque, $\tau$, defined in Eq. 22:

22 $$\begin{aligned} \tau =\left( \frac{\pi ^2ka}{16}\right) \frac{1}{E_0|\Omega |}<T_z> \end{aligned}$$

In Eq. 22, $E_0=\frac{1}{2}\rho _0\mathcal {V}_0^2$ is the characteristic kinetic energy of the self-radiating cylinder and $\Omega =\pi a^2L$ is its volume.

## Results and discussion

### Sample self-rotating cases

**Fig. 3:**
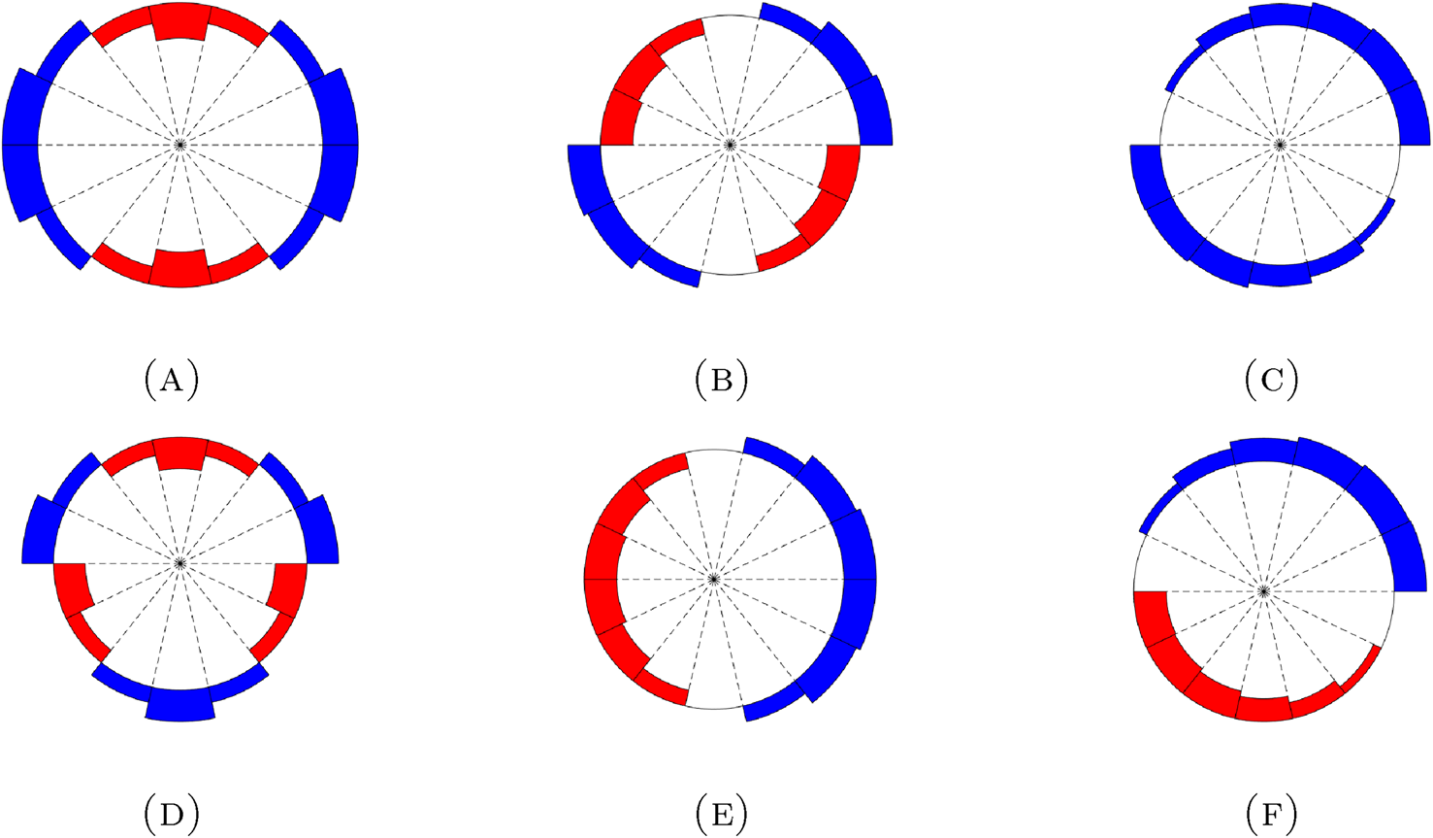
Illustration of six boundary conditions $\mathcal {V}(\theta )$ (See Eq. 20) corresponding to (a) Case 2$\pi$-CS, (b) Case $\pi$-CS, (c) Case $\frac{\pi }{2}$-CS, (d) Case 2$\pi$-CAS, (e) Case $\pi$-CAS, (f) Case $\frac{\pi }{2}$-CAS.

To examine more tangibly the self-rotation phenomenon for a vibrating cylidner, we start by calculating $\tau$ for terminal phases $\phi _0=2\pi ,\pi$, and $\frac{\pi }{2}$ applied to CS and CAS phase distributions. We denote these special cases by Case 2$\pi$-CS, Case $\pi$-CS, Case $\frac{\pi }{2}$-CS, Case $2\pi$-CAS, Case $\pi$-CAS, and Case $\frac{\pi }{2}$-CAS respectively. The imposed boundary velocity for each sample case is shown in Fig. 3a to 3f. In these figures, $N_h=7$ is assumed for illustration purposes.

**Fig. 4:**
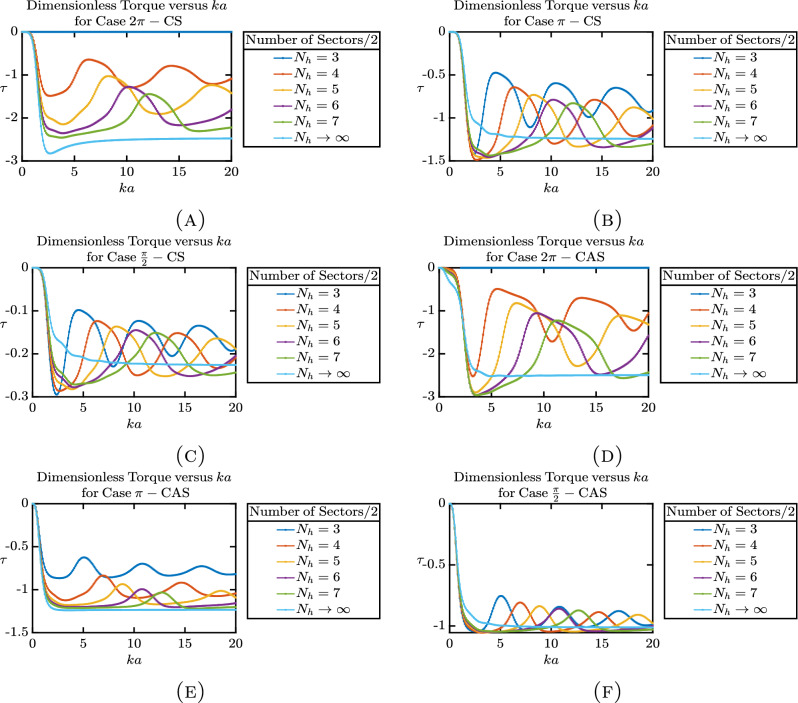
Values of dimensionless radiation torque plotted for $0\le ka\le 20$ with a resolution of 0.1 and different number of sectors. (a), (b), and (c) CS phase distributions with their terminal phases equal to $2\pi$, $\pi$ and $\frac{\pi }{2}$, respectively. (d), (e), and (f) CAS phase distributions with their terminal phases equal to $2\pi$, $\pi$ and $\frac{\pi }{2}$, respectively.

The radiation torque in each case is evaluated using Eq. 22 for number of sectors $N=2N_h=6,8,10,12$, and 14, and for nondimensional wavenumber, *ka*, ranging from 0.1 to 20 with a resolution of 0.1. The results are presented in Fig. 4a-4f for each of the sample cases (Table 1).

**Table 1:** Physical constants used in the numerical calculations.

Physical constant	Description	Value
$c_0$	Speed of sound	$1500 (\frac{\text {m}}{\text {s}})$
$\rho _0$	Fluid density	$997 (\frac{\text {kg}}{\text {m}^3})$
*a*	Cylinder’s radius	$0.001 (\text {m})$
*ka*	Dimensionless wavenumber	$0.1-20$
$\mathcal {V}_0$	Maximum vibration amplitude of $\partial \Omega$	$0.001\>(\frac{\text {m}}{\text {s}})$
$E_0$	Characteristic kinetic energy	$\text {4.985e-4}\> (\frac{\text {J}}{\text {m}^3})$
$\Omega$	Cylinder’s volume	$\text {3.1416e-6}\> (\text {m}^3)$

The acoustic radiation torque vanishes for the Case $2\pi$-CS when $2N_h=6$ (Fig. 4a). This happens due to the velocity phases over each sector being either $0,\pi$, or $2\pi$ which forces the sectors to vibrate either in-phase or antiphase. According to Eq. 19, such a phase distribution results in no net radiation torque. Going through Fig. 4a-4f, the amplitude of the radiation torque is, in a general sense, larger in magnitude for the configurations with more number of sectors. The limiting case is when $N_h\rightarrow \infty$, in which the oscillating behavior of $\tau$ has faded away, allowing the radiation torque to reach a constant value for $ka\gtrapprox 5$ in Cases $2\pi$- and $\pi$-CAS, and $ka\gtrapprox 10$ for the remaining cases. Subsequently, we can infer that $\tau \sim 1\Rightarrow <T_z>\sim \frac{1}{ka}$ according to Eq. 22. From a design point of view, increasing the number of sectors in a self-rotating cylinder will result in radiation torques that are more robust to variations in frequency, as shown in Fig. 4a to 4f ($N_h=200$ is set in the following calculations to approximate $N_h\rightarrow \infty$).

**Fig. 5:**
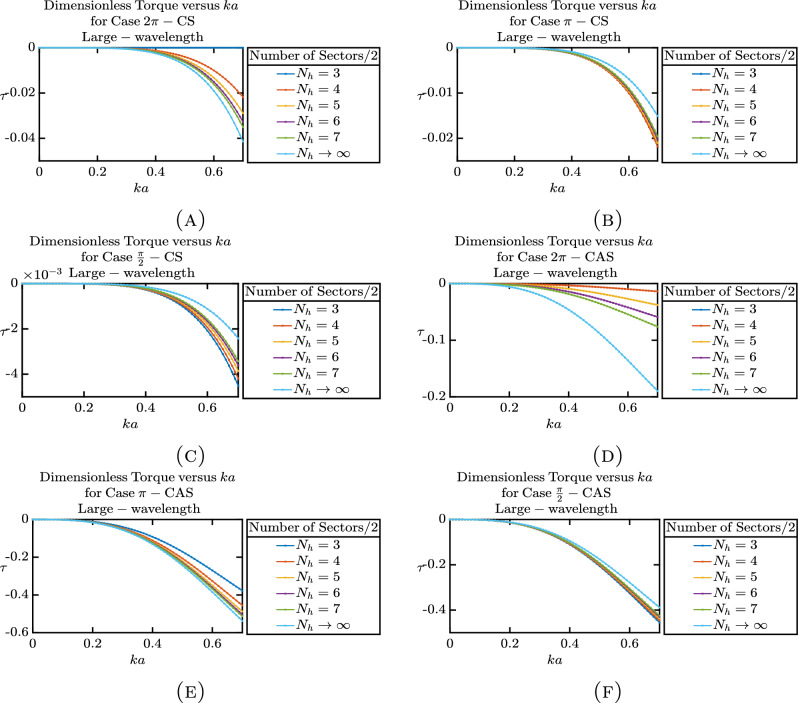
Values of dimensionless radiation torque plotted for $0.01\le ka\le 0.7$ with a resolution of 0.01 and different number of sectors. (a), (b), and (c) CS phase distributions with their terminal phases equal to $2\pi$, $\pi$ and $\frac{\pi }{2}$, respectively. (d), (e), and (f) CAS phase distributions with their terminal phases equal to $2\pi$, $\pi$ and $\frac{\pi }{2}$, respectively.

The large-wavelength regime $(ka<0.7)$ is studied in more detail due its importance in microparticle dynamics in acoustic manipulation. The radiation torque in this region is evaluated for a range of *ka* from 0.01 to 0.7 with a resolution of 0.01. The results are shown in Fig. 5a to 5f, respectively.

In the long-wavelength regime the nondimensional torque $\tau$ is controlled by the lowest non-vanishing angular order:

23 $$\begin{aligned} n^\star \;=\; \min \{\,n\ge 1:\;S_n\ne 0\,\},\qquad S_n:=\sum _{1\le i<j\le N}\sin (\phi _i-\phi _j)\,\Lambda _{n,i,j}, \end{aligned}$$

i.e., the first *n* for which the phase–geometry sum $S_n$ does not cancel. Using the small-argument expansion $\frac{1}{\big |H_n'(ka)\big |^2}\;\sim \; \text {(const)}\,(ka)^{2n+2}$ and our normalization in Eq. 22, the $n^{th}$ summand scales as

24 $$\begin{aligned} \tau _n(ka)\;\propto \; S_n\, (ka)^{2n+1},\qquad ka\ll 1 \end{aligned}$$

and consequently,

25 $$\begin{aligned} \tau (ka)\;\propto \; (ka)^{2n^{\star }+1},\qquad ka\ll 1 \end{aligned}$$

These small-*ka* behaviors dovetail with the multipolar viewpoint emphasized by^40^: in the long-wavelength limit the first non-vanishing angular order dictates the power law of the radiation torque. Our sectorized boundary acts as a tunable source that either admits or suppresses low orders via its phase pattern, so the measured slope $2n^{\star }+1$ becomes a direct “fingerprint” of the phase geometry. In particular, patterns that continuously wind the phase (our CAS cases) leave $n^{\star }=1$ active and yield $\tau \sim (ka)^3$, whereas discretizations that enforce pairwise antiphase relationships may eliminate the dipole and push the response to $(ka)^5$ (or higher) until symmetry is broken. Note that in the long-wavelength regime, the absolute torque is tiny (by Eq. 25), so small modeling or truncation effects (meshing of sector jumps, outer-domain/PML tuning, or omission of thermoviscous layers) can translate into substantial relative deviations even when the asymptotic slope and the overall trend are correct. This sensitivity vanishes as *ka* grows and the field becomes more radiative, which is consistent with the sub-percent agreement we observe for $ka>1$, as seen in Section Numerical validation.

This section ends with a note on the long-wavelength regime. The two-dimensional (infinite-length) model employed throughout this work is exact when $L\rightarrow \infty$. For finite cylinders in the long-wavelength regime, the 2D approximation remains valid provided $\beta =L/a\gg 1$ (negligible axial variation) and $kL\gg 1$ (minimal end backscattering). These conditions combine to give the practical requirement

$$\beta \gg \frac{1}{ka}.$$

The most stringent case occurs in the large-wavelength regime considered in Fig. 5. For ($ka=0.1$) we have $\beta \gg 10$ or $L>100a$. A cylinder of radius $a=10$ $\mu m$ then corresponds to $L\gtrsim 1$ mm. While such an aspect ratio might not be easy to manufacture, the predicted pure rotational motion (zero net force, non-zero torque) and the scaling $\tau (ka)\propto (ka)^{2n^*+1}$ remain physically valid. For even larger wavelengths, the aforementioned scale analysis provides a lower-bound for the length of the cylinder, given the design is based on Eq. 21.

### Numerical validation

**Fig. 6:**
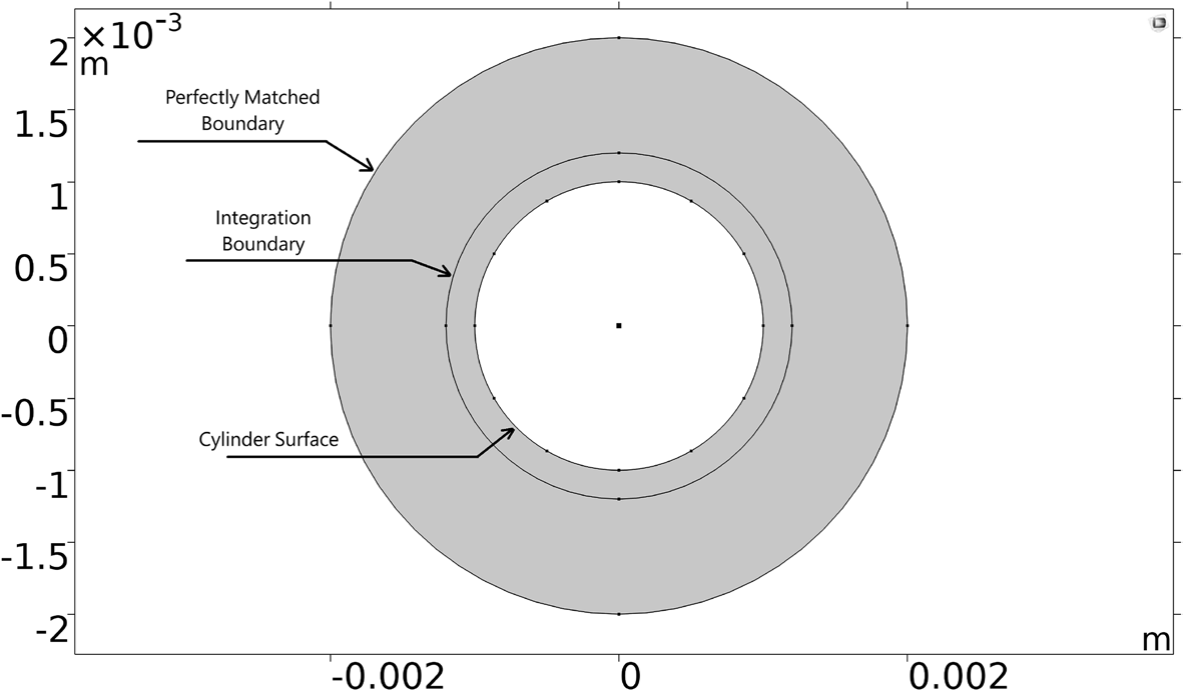
Configuration of the problem in COMSOL.

Eq. 21a and 21b for acoustic radiation torque are numerically validated by simulating the six sample cases proposed in Section Sample self-rotating cases via finite element analysis. The Pressure Acoustics physics of the COMSOL Multiphysics^®^ software is used for this purpose. The problem is assumed to be 2D which represents a cylinder of infinite length. A circle with radius $a=1\textrm{ mm}$ is plotted that models the cylinder’s surface, $\partial \Omega$. A second circle with a radius of $1.2\textrm{ mm}$ is used as the imaginary boundary over which the integral of radiation torque (Eq. 19) is computed. The infinity radiation condition is modeled by a third circle with a radius equal to $2\textrm{ mm}$, which ensures that no waves would be reflected back to the cylinder. It should be noted that the integration boundary can be selected arbitrarily as a direct result of Eq. 16. Moreover, care must be taken to ensure that the selected perfectly matched layer does not allow any reflections back into the model. To make sure, mesh convergence was assessed by systematically increasing the elements-per-wavelength (EPW) parameter from 5 to 20, with a step size of 5. Convergence in radiation torque was confirmed for all sample cases and wavenumbers by using a relative error index, with all values falling below 1%. Fig. 6 illustrates the problem as modeled in COMSOL Multiphysics^®^.

The speed of sound and the density of the medium are given according to Section Sample self-rotating cases. The mesh settings are physics-controlled, and a mesh convergence study prompted to use 20 elements per wavelength to ensure convergence for all simulations, which demanded an average number of 110000 quadratic triangular elements. Note that the convergence metric in all simulations was the relative error in $\tau$ in two consecutive mesh refinements. Normal Velocity boundary conditions are imposed on the cylinder’s surface, while a Perfectly Matched Boundary Condition is imposed on the outer surface of the cylinder with 80 mesh points.

Although the radiation torque is not automatically calculated, it can be readily post-processed by first calculating the radial and angular components of the velocity field that is calculated in its Cartesian form inside the software. The radiation torque integral in Eq. 19 is then approximated over $\partial \Omega _{\infty }$ by interpolating the velocity field using the shape functions of the finite elements that comprise $\partial \Omega _{\infty }$. We define $\delta _{\tau }$ as the relative error of the analytically derived radiation torque, $\tau _a$, with respect to the corresponding results of the finite element analysis, $\tau _{\text {FEA}}$:

26 $$\begin{aligned} \delta _{\tau }=|\frac{\tau _a-\tau _{\text {FEA}}}{\tau _{\text {FEA}}}|\times 100\> (\%) \end{aligned}$$

**Fig. 7:**
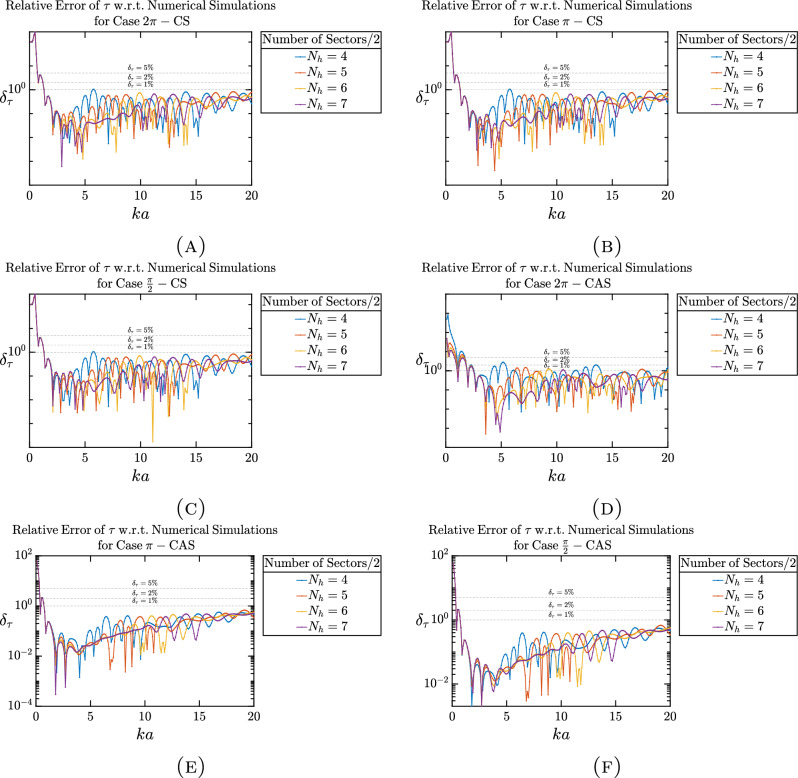
Illustration of relative error in calculation of acoustic radiation torque for (a) Case 2$\pi$-CS, (b) Case $\pi$-CS, (c) Case $\frac{\pi }{2}$-CS, (d) Case 2$\pi$-CAS, (e) Case $\pi$-CAS, (f) Case $\frac{\pi }{2}$-CAS.

Fig. 7a to 7f illustrate the values of $\delta _{\tau }$ for the six cases 2$\pi$-CS, $\pi$-CS, $\frac{\pi }{2}$-CS, $2\pi$-CAS, $\pi$-CAS, and $\frac{\pi }{2}$-CAS, respectively.

It should be noted that the case of $N_h=3$ is excluded from the results of Fig. 7a to 7f for the terminal phase $\phi _0=2\pi$, as they evaluate to zero radiation torque. It can be inferred from Fig. 7a to 7f that the analytical formula derived for the acoustic radiation torque in Eq. 21a and 21b are correct and show a remarkable consistency with numerical simulations, with $\delta _{\tau }<1\%$ for the majority of cases. However, the relative error exceeds $5\%$ in the long-wavelength regime ($ka<0.7$). This is due to the round-off errors in the calculation of inverse derivatives of the Hankel function in Eq. 21a and 21b, as well as the high sensitivity of radiation torque to the location of perfectly matched layer^41^.

For the long-wavelength regime, the radius of the perfectly matched layer is set as $\lambda _{max}=\frac{c_0}{f_{min}}\sim 0.6 (\text {m})$ where the minimum frequency in the simulation is set as $f_{min}=2387.3 (\text {Hz})$ corresponding to $ka=0.01$. A resolution of 0.01 is adopted to sweep all wavenumbers between 0.01 to 0.7. This improves the consistency between analytical and numerical results to more than 90% for all cases.

### Radiation torque vs. the number of sectors

**Fig. 8:**
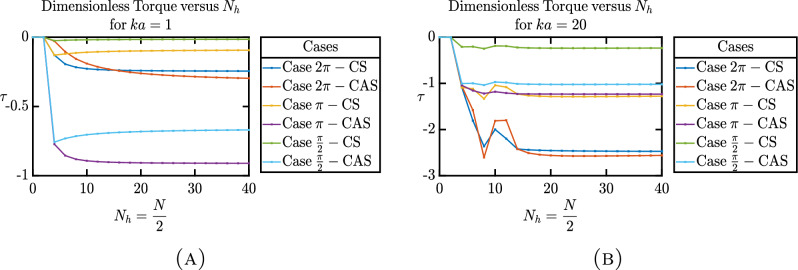
Convergence of dimensionless radiation torque as the number of sectors increase plotted for (a) $ka=1$, (b) $ka=20$.

The relation between the acoustic radiation torque and the number of sectors can be analyzed in more detail. We calculate $\tau$ for sector numbers ranging from $2N_h=2,4,\cdots ,80$ for cases $ka=1$ and $ka=20$ in Fig. 8a and 8b, respectively.

Fig. 8a and 8b suggest that the value of the radiation torque converges to constant values for all studied cases, regardless of the dimensionless wavenumber. However, the number of sectors required for stabilization of $\tau$ increases for larger values of *ka*. This can be explained by the behavior of the $\frac{ka}{|{H_n^{(1)}}^{'}(ka)|^2}$, which experiences its maximum value around $ka=n$. In order for the convergence to happen, $N_h$ must be large enough such that $ka=n<<N_h$. More can be said for the behavior of the ART when $N_h\rightarrow \infty$. According to Eq. 12, the Fourier coefficients $|A_n|$ and $|B_n|$ decay like $\mathcal {O}(\frac{1}{n})$, implying $n|A_n^*B_n|\sim \mathcal {O}(\frac{1}{n})$. Each summand of Eq. 22 therefore behaves accordingly, allowing us to truncate the series in a finite $n=n_{\text {tr}}$. The limits $N_h\rightarrow \infty$ and $\frac{n}{N_h}\rightarrow 0$ then become equivalent, and we have:

27 $$\begin{aligned} \begin{aligned}&\text {CS distribution:}\\&\lim _{N_h\rightarrow \infty }\tau \sim \frac{4}{ka}\sum _{n=2,4,\cdots }^{n_{\text {tr}}}\frac{-\sin ^2\left( \frac{\phi _0}{2}\right) \phi _0n^2\pi ^2}{(\phi _0^2-n^2\pi ^2)^2|{H_n^{(1)}}^{'}(ka)|^2}{\mathop {\sim }\limits ^{ka\rightarrow \infty }}-\frac{\pi }{8}(\phi _0-\sin (\phi _0))\\&\text {CAS distribution:}\\&\lim _{N_h\rightarrow \infty }\tau \sim \frac{4}{ka}\sum _{n=1,3,\cdots }^{n_{\text {tr}}}\frac{-\cos ^2\left( \frac{\phi _0}{2}\right) \phi _0n^2\pi ^2}{(\phi _0^2-n^2\pi ^2)^2|{H_n^{(1)}}^{'}(ka)|^2}{\mathop {\sim }\limits ^{ka\rightarrow \infty }}-\frac{\pi }{8}(\phi _0+\sin (\phi _0))\\ \end{aligned} \end{aligned}$$

Eq. 27 yields $-1.00955$ and $-0.224151$ for cases $\frac{\pi }{2}$-CAS and $\frac{\pi }{2}$-CS, yields $-1.2337$ for cases $\pi$-CS and $\pi$-CAS, and yields $-2.4674$ for cases $2\pi$-CS and $2\pi$-CAS. These values are consistent with Fig. 8b.

### Radiation torque vs. the nondimensional wavenumber and the terminal phase

**Fig. 9:**
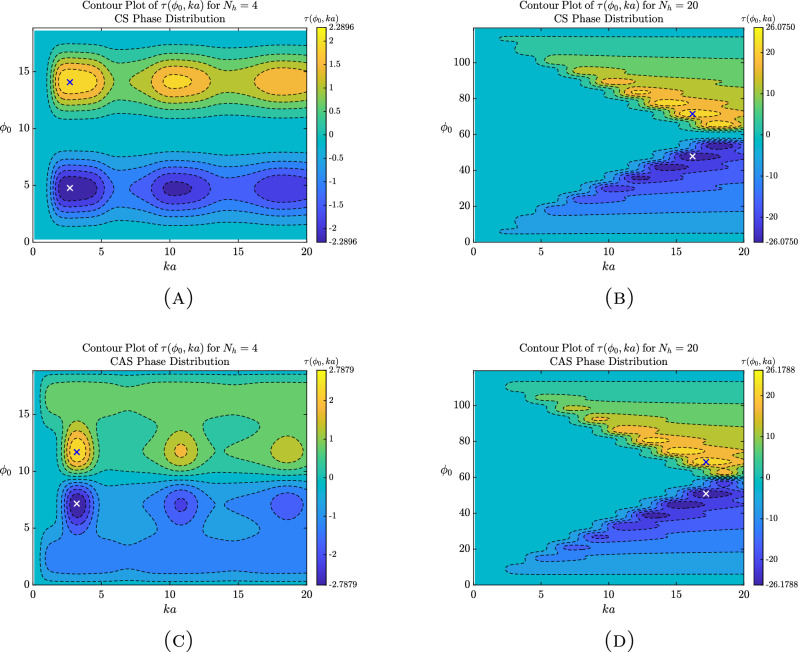
The acoustic radiation torque computed for $0\le ka\le 20$ and $0\le \phi _0\le 2(N_h-1)\pi$ assuming (a) $N_h=4$ and a CS phase distribution, (b) $N_h=20$ and a CS phase distribution, (c) $N_h=4$ and a CAS phase distribution, and (d) $N_h=20$ and a CAS phase distribution. The maximum and minimum values of $\tau$ are provided in the colorbar and their locations are marked with blue and white crosses, respectively.

Finally, we investigate the behavior of the ART as a function of the nondimensional wavenumber *ka* and the terminal phase $\phi _0$. Fig. 9a and 9b demonstrate the behavior of $\tau (ka,\phi _0)$ for $N_h=4$ and $N_h=20$ respectively in a CS phase distribution, and Fig. 9c and 9d present $\tau (ka,\phi _0)$ in similar settings for a CAS phase distribution.

The magnitude of the radiation torque has a periodicity of $2(m-1)\pi$ in $\phi _0$, according to Fig. 9, and changes sign at $\phi _0=(m-1)\pi$. More than $\times 11$ increase in $\tau$ is obtained by using 40 instead of 8 sectors for the cylinder. The maximum and minimum values of $\tau$ are marked with blue and white crosses respectively in Fig. 9.

Finally, the angular velocity achievable by our proposed self-rotating mechanism is computed. Assuming that the hydrodynamic and acoustic physics are decoupled and that the inertial effects are negligible in the hydrodynamic regime (See Supplementary Eq. S26 to S29 for details), the radiation torque and the consequent Stokes drag result in a terminal angular velocity^42^:

28 $$\begin{aligned} T_{\text {drag}}=<T_z>=4\pi \mu \omega _{\text {spin}}a^2L\Rightarrow \omega _{\text {spin}}=\frac{2\rho _0\mathcal {V}_0^2}{\pi ^2\nu }\tau \end{aligned}$$

where $\omega _{\text {spin}}$ is the terminal, steady angular velocity of the self-rotating cylinder, and $\nu$ is the dynamic viscosity of the fluid. Let $\rho _0\sim \mathcal {O}(10^3)\text {kg}/\text {m}^3$, $L\sim \mathcal {O}(10^{-2})\text {m}$, $a\sim \mathcal {O}(10^{-3})\text {m}$, and $\mu _{\text {dy}}\sim \mathcal {O}(10^{-3})\text {Pa.s}$ be their orders of magnitude. Define the hydrodynamic Reynolds number as

29 $$\begin{aligned} \text {Re}=\frac{\rho _0uL}{\nu }=\frac{\rho _0\omega _{\text {spin}}aL}{\nu } \end{aligned}$$

For the Reynolds number to remain in the creeping flow regime, i.e. $\text {Re}\sim \mathcal {O}(10^{-1})$, we should have $\omega _{\text {spin}}\lesssim \mathcal {O}(10^{-2})\text {rad/s}$. A typical velocity amplitude of $\mathcal {V}_0\sim \mathcal {O}(10^{-3})\text {m/s}$ will therefore ensure the low Reynolds number assumption by keeping the angular velocity around $\mathcal {O}(10^{-1})$ to $\mathcal {O}(10^{-2})$ for $ka\sim \mathcal {O}(1)$ and $ka\sim \mathcal {O}(10^{1})$, respectively. This implies that greater frequencies allow better stability but lower rotation velocities for a self-rotating design.

## Conclusion

The concept of acoustical self-rotating cylinders is introduced in this paper. To achieve self-rotation, the boundary of the cylinder is partitioned into sectors that radiate with equal amplitude but unequal phases. Assuming axisymmetry with respect to the out-of-plane axis, the wave equation is analytically solved and the acoustic radiation force and torque are reproduced in terms of the scattering coefficients. Two general families of phase distributions across the cylinder’s boundary are proved to result in a zero net motion but non-zero rotation, due to the nonlinear transfer of angular momentum flux from the stimulated fluid to the cylinder. Simplified equations are derived for the case of equiangular sectors and linearly varying phase distributions, allowing a better understanding of the optimum design parameters, as well as the asymptotic behavior of the radiation torque for limiting frequencies and partitionings. The derived equations were further validated numerically by conducting finite element analyses for all cases and number of sectors.

It was shown that the value of the terminal phase has a substantial influence on the magnitude of the radiation torque, maximizing it for one of the finitely many phases $\phi _0=\frac{k(N-2)\pi }{N}$ where $1\le k\le \frac{N}{2}\in \mathbb {N}$ and *N* is the number of sectors used. The wave-like behavior of acoustic radiation torque fades away for a large enough number of sectors ($N\ge 2ka$), making it a desirable design goal for acquiring stable rotations in the presence of fluctuations in the radiation frequency. Increasing the number of sectors also dramatically enlarges the space of feasible radiation torques; for example, from $\tau \in [-2.7,2.7]$ for $N=8$ to $\tau \in [-26.1,26.1]$ for $N=40$. Finally, it is argued that for typical values of radiation amplitude $\mathcal {V}_0\sim \mathcal {O}(10^{-3})\text {m/s}$ and frequency $\omega \sim \mathcal {O}(10^6)$, promising values of angular velocities in the order of $\mathcal {O}(10^{-1})\text {rad/s}$ can be obtained while staying in the low Reynolds number regime.

From a critical point of view, this paper presents its formulation only to prove the feasibility of the concept. Importantly, the nature of the excitations and the fabrication means to achieve it need to be addressed. The prescribed boundary excitation can be realized with ultrathin piezoelectric films (e.g., PVDF or thin-film PZT) wrapped as a ring and segmented into angular sectors over a common inner electrode. Equal-amplitude, phase-shifted voltage drives applied to the sectors in thickness mode impose the required normal surface velocity, and the assembly can be conformally encapsulated for underwater use. Such sectorized flexible transducers and multichannel phase control are well established, providing a practical route to experimental validation. The simplicity of the design, as well as the derived mathematical equations that directly link the radiation torque with the design parameters, are expected to pave the way toward designing miniaturized acoustic mechanisms to be employed in various medical and engineering domains.

## Supplementary Information

Supplementary Information.

## Data Availability

Data sets generated during the current study are available from the corresponding author on reasonable request.
