# Supplementary material for: Acoustic-driven self-rotating cylinders
Source: Sci Rep. 2026 May 6;16:20812. doi: 10.1038/s41598-026-51486-y (PMC13338191; doi:10.1038/s41598-026-51486-y)
Supplement: Supplementary file 1 — Supplementary Information. [file 41598_2026_51486_MOESM1_ESM.pdf]

Supplementary Information

Derivation of Acoustic Radiation Force and Torque

In this section, the full derivations of acoustic radiation force and torque (Eq. 2.17 and 2.19) are provided.

Derivation of Acoustic Radiation Force

Step 0 — Notation. For sector j with endpoints $(\zeta_{2j-1}, \zeta_{2j})$, define its midpoint and width

$$\zeta_j^* = \frac{\zeta_{2j-1} + \zeta_{2j}}{2}, \qquad \Delta\zeta_j = \zeta_{2j} - \zeta_{2j-1}. \tag{S1}$$

Step 1 — Scattering coefficients A_n, B_n. Assume the angular expansion of the velocity potential

$$\psi(r,\theta) = \psi_0 \sum_{n=0}^{\infty} \big(A_n \sin n\theta + B_n \cos n\theta\big)\, H_n^{(1)}(kr). \tag{S2}$$

Imposing the Neumann boundary condition for N sectors vibrating with equal amplitude V_0 and phases $\{\phi_j\}$ gives, for $n \geq 1$,

$$B_n = \frac{V_0}{n\pi k\, \psi_0\, H_n^{(1)\prime}(ka)} \sum_{j=1}^{N} e^{\mathrm{i}\phi_j} \Big[\sin\big(n\zeta_{2j}\big) - \sin\big(n\zeta_{2j-1}\big)\Big], \tag{S3}$$

$$A_n = \frac{V_0}{n\pi k\, \psi_0\, H_n^{(1)\prime}(ka)} \sum_{j=1}^{N} e^{\mathrm{i}\phi_j} \Big[\cos\big(n\zeta_{2j-1}\big) - \cos\big(n\zeta_{2j}\big)\Big]. \tag{S4}$$

Using

$$\sin(n\beta) - \sin(n\alpha) = 2\cos\Big(n\frac{\alpha+\beta}{2}\Big)\sin\Big(n\frac{\beta-\alpha}{2}\Big)$$

and

$$\cos(n\alpha) - \cos(n\beta) = 2\sin\Big(n\frac{\alpha+\beta}{2}\Big)\sin\Big(n\frac{\beta-\alpha}{2}\Big),$$

define the sector "atoms"

$$b_{n,j} = 2\cos(n\zeta_j^*)\sin\Big(\frac{n\,\Delta\zeta_j}{2}\Big), \qquad a_{n,j} = 2\sin(n\zeta_j^*)\sin\Big(\frac{n\,\Delta\zeta_j}{2}\Big), \tag{S5}$$

and write

$$A_n = C_n \sum_{j=1}^{N} e^{\mathrm{i}\phi_j} a_{n,j}, \qquad B_n = C_n \sum_{j=1}^{N} e^{\mathrm{i}\phi_j} b_{n,j}, \qquad C_n := \frac{V_0}{n\pi k\, \psi_0\, H_n^{(1)\prime}(ka)}. \tag{S6}$$

(For $n = 0$ we have $A_0 = 0$; the $n = 0$ contribution to the force below vanishes due to a prefactor $n(n+1)$.)

Step 2 — Structure of the acoustic radiation force (their Eq. 2.17). After the standard far-field reduction one obtains

$$\langle \mathbf{F} \rangle = -\rho_0 \frac{\psi_0^2}{k} L \sum_{n,m\geq 0} \mathrm{Re}\Big\{ \mathrm{i}^{\,m-n} \Big[\gamma_{n,m} \big(A_n A_m^* + B_n B_m^* \big) \,\hat{\mathbf{i}} + \lambda_{n,m} \big(A_n B_m^* - B_n A_m^* \big) \,\hat{\mathbf{j}} \Big] \Big\}\,, \tag{S7}$$

where $\gamma_{n,m}$ and $\lambda_{n,m}$ contain only $\delta_{m,n\pm1}$, so only adjacent modes couple. Reindex to pair n with $n{+}1$.

Step 3 — Expand sector sums and separate phases. Using the expressions for A_n, B_n,

$$A_n A_{n+1}^* + B_n B_{n+1}^* = C_n C_{n+1}^* \sum_{j,k=1}^{N} e^{\mathrm{i}(\phi_j - \phi_k)} \big(a_{n,j} a_{n+1,k} + b_{n,j} b_{n+1,k} \big), \tag{S8}$$

$$A_n B_{n+1}^* - B_n A_{n+1}^* = C_n C_{n+1}^* \sum_{j,k=1}^{N} e^{\mathrm{i}(\phi_j - \phi_k)} \big(a_{n,j} b_{n+1,k} - b_{n,j} a_{n+1,k} \big). \tag{S9}$$

Using $H^{(2)} = \overline{H^{(1)}}$ for real ka and collecting constants yields the compact form

$$\langle \mathbf{F} \rangle = \frac{4\rho_0 L V_0^2}{\pi^2 k} \sum_{n=0}^{\infty} \sum_{j=1}^{N} \sum_{k=1}^{N} \mathrm{Im}\Big\{ e^{\mathrm{i}(\phi_j - \phi_k)}\, n(n{+}1)\, H_n^{(1)\prime}(ka)\, H_{n+1}^{(2)\prime}(ka) \Big\}\; \boldsymbol{\Upsilon}_{n,j,k}\,, \tag{S10}$$

where $\boldsymbol{\Upsilon}_{n,j,k}$ is purely geometric (depends only on the ζ's).

Step 4 — Evaluate the geometry vector $\boldsymbol{\Upsilon}_{n,j,k}$. Using the formulae

$$\cos x \cos y + \sin x \sin y = \cos(x - y)$$

and

$$\sin x \cos y - \cos x \sin y = \sin(x - y),$$

we find

$$a_{n,j} a_{n+1,k} + b_{n,j} b_{n+1,k} = 4 \sin\Big(\frac{n \Delta\zeta_j}{2} \Big) \sin\Big(\frac{(n{+}1)\Delta\zeta_k}{2} \Big) \cos\Big(n\zeta_j^* - (n{+}1)\zeta_k^* \Big), \tag{S11}$$

$$a_{n,j} b_{n+1,k} - b_{n,j} a_{n+1,k} = 4 \sin\Big(\frac{n \Delta\zeta_j}{2} \Big) \sin\Big(\frac{(n{+}1)\Delta\zeta_k}{2} \Big) \sin\Big(n\zeta_j^* - (n{+}1)\zeta_k^* \Big). \tag{S12}$$

Therefore, for $n \geq 1$,

$$\boldsymbol{\Upsilon}_{n,j,k} = 4\, \sin\Big(\frac{n\, \Delta\zeta_j}{2} \Big)\, \sin\Big(\frac{(n{+}1)\, \Delta\zeta_k}{2} \Big)\, \begin{bmatrix} \cos\big(n\, \zeta_j^* - (n{+}1)\, \zeta_k^* \big) \\ \sin\big(n\, \zeta_j^* - (n{+}1)\, \zeta_k^* \big) \end{bmatrix}. \tag{S13}$$

(The $n = 0$ term in the force sum vanishes due to the prefactor $n(n{+}1)$.)

Step 5 — Equi-angular sectors (useful special case). If the cylinder is split into $N = 2N_h$ equal sectors,

$$\Delta\zeta_r = \frac{\pi}{N_h}, \qquad \zeta_r^* = \frac{(2r-1)\pi}{2N_h} \quad (r = 1, \ldots, N), \tag{S14}$$

then

$$\boldsymbol{\Upsilon}_{n,j,k} = 4\,\sin\Bigl(\frac{n\pi}{2N_h}\Bigr)\,\sin\Bigl(\frac{(n{+}1)\pi}{2N_h}\Bigr)\begin{bmatrix}\cos\Bigl(\frac{n(2j-1)-(n+1)(2k-1)}{2N_h}\pi\Bigr)\\ \sin\Bigl(\frac{n(2j-1)-(n+1)(2k-1)}{2N_h}\pi\Bigr)\end{bmatrix}. \tag{S15}$$

Derivation of Acoustic Radiation Torque

Step 1 — Far-field radiation torque. Using the angular-momentum flux identity, the time-averaged z-component of the radiation torque for an infinite cylinder can be written as

$$\bigl\langle T_z \bigr\rangle \;=\; 2\,\rho_0\,L\,\psi_0^2\sum_{n=1}^{\infty} n\;\mathrm{Im}\bigl\{A_n^* B_n\bigr\}, \tag{S16}$$

where A_n and B_n are the scattering coefficients.
Step 2 — Sectorized boundary. For a boundary partitioned into N sectors with equal normal velocity amplitude V_0 and phases $\{\phi_i\}_{i=1}^{N}$, and defining each sector's midpoint and width as

$$\zeta_i^* = \frac{\zeta_{2i-1} + \zeta_{2i}}{2}, \qquad \Delta\zeta_i = \zeta_{2i} - \zeta_{2i-1}, \tag{S17}$$

the coefficients (for $n \geq 1$) read

$$B_n = \frac{V_0}{n\pi k\,\psi_0\,H_n^{(1)\prime}(ka)}\sum_{i=1}^{N} e^{\mathrm{i}\phi_i}\Bigl[\sin\bigl(n\zeta_{2i}\bigr) - \sin\bigl(n\zeta_{2i-1}\bigr)\Bigr], \tag{S18}$$

$$A_n = \frac{V_0}{n\pi k\,\psi_0\,H_n^{(1)\prime}(ka)}\sum_{i=1}^{N} e^{\mathrm{i}\phi_i}\Bigl[\cos\bigl(n\zeta_{2i-1}\bigr) - \cos\bigl(n\zeta_{2i}\bigr)\Bigr]. \tag{S19}$$

Step 3 — Isolate phase vs. geometry in $\mathrm{Im}\{A_n^* B_n\}$. Introduce the sector "atoms" from Eq. S5 and the prefactor C_n from Eq. S6. Form the product $\mathrm{Im}\{A_n^* B_n\}$ and take the imaginary part by pairing (i,j) and (j,i) terms:

$$A_n^* B_n = |C_n|^2 \sum_{i,j} e^{\mathrm{i}(\phi_j - \phi_i)}\,a_{n,i}\,b_{n,j}, \tag{S20}$$

$$\mathrm{Im}\{A_n^* B_n\} = |C_n|^2 \sum_{1\leq i<j\leq N} \sin(\phi_i - \phi_j)\,\bigl(a_{n,j}b_{n,i} - a_{n,i}b_{n,j}\bigr). \tag{S21}$$

Using the trigonometric identities with Eq. S5,

$$a_{n,j}b_{n,i} - a_{n,i}b_{n,j} = 4\sin\Bigl(\frac{n\Delta\zeta_j}{2}\Bigr)\sin\Bigl(\frac{n\Delta\zeta_i}{2}\Bigr)\sin\bigl(n(\zeta_j^* - \zeta_i^*)\bigr). \tag{S22}$$

Step 4 — Collect constants to match Eq. (2.19) and read off $\Lambda_{n,i,j}$. Since $|C_n|^2 = \dfrac{V_0^2}{n^2\pi^2k^2\,|H_n^{(1)\prime}(ka)|^2}$, substitute into (S16) to obtain

$$\langle T_z\rangle = \frac{8\,\rho_0\,L\,V_0^2}{\pi^2k^2}\sum_{n=1}^{\infty}\frac{1}{n\,\left|H_n^{(1)\prime}(ka)\right|^2}\sum_{1\le i<j\le N}\sin(\phi_i-\phi_j) \\ \times\sin\big(n(\zeta_j^*-\zeta_i^*)\big)\,\sin\Big(\frac{n\Delta\zeta_j}{2}\Big)\,\sin\Big(\frac{n\Delta\zeta_i}{2}\Big). \tag{S23}$$

Thus, the *geometry-only* factor (last part of Eq. (2.19)) is

$$\Lambda_{n,i,j} = \sin\big(n(\zeta_j^*-\zeta_i^*)\big)\,\sin\Big(\frac{n\Delta\zeta_j}{2}\Big)\,\sin\Big(\frac{n\Delta\zeta_i}{2}\Big), \qquad (n\ge 1). \tag{S24}$$

It is immediately clear that $\Lambda_{n,i,j} = -\Lambda_{n,j,i}$, so the pair sum can be written with $i<j$ and $\sin(\phi_i-\phi_j)$ as above.

Equi-angular special case. If $N = 2N_h$ equal sectors, then $\Delta\zeta_i = \pi/N_h$ and $\zeta_i^* = \frac{(2i-1)\pi}{2N_h}$, giving

$$\Lambda_{n,i,j} = \sin\Big(\frac{n(j-i)\pi}{N_h}\Big)\,\sin^2\Big(\frac{n\pi}{2N_h}\Big). \tag{S25}$$

Scale Separation Between Hydrodynamics and Acoustics

To show that the hydrodynamic and acoustic physics are decoupled, the first step is to analyze the time-scales of these two physics. Denote by t_a, l^*, V_0 and p^* the acoustic time scale, the characteristic length, the characteristic velocity, and the characteristic pressure so that $t = t_a\bar{t}$, $(x,y,z) = l^*(\overline{x},\overline{y},\overline{z})$, $\boldsymbol{v} = V_0\overline{\boldsymbol{v}}$, and $p = p^*\overline{p}$. Equation 2.2b can then be rewritten as

$$\frac{\rho_0 V_0 l^*}{t_a p^*}\frac{\partial\overline{\boldsymbol{u}}}{\partial\overline{t}} = -\nabla\overline{p} \tag{S26}$$

Consequently, $t_a \sim \frac{\rho_0 V_0 l^*}{p^*}$. Moreover, according to Eq. 2.4:

$$p^*\overline{p} = {c_0}^2\rho = {c_0}^2\epsilon\mathcal{O}(\rho_0) \sim c_0V_0\rho_0 \Rightarrow t_a \sim \frac{l^*}{c_0} \tag{S27}$$

where $\epsilon = \frac{U^*}{c_0}$ is the acoustic perturbation factor. For the hydrodynamic physics where the time-averaged radiation torque is applied as an external load, low Reynolds number condition is assumed, which is a common assumption in acoustofluidics [1]. The aforementioned assumption gives the hydrodynamic time scale equal to $t_s = \frac{{l^*}^2}{\nu}$ [2]. Accordingly, the ratio of these time scales results in the Reynolds number

$$\frac{t_s}{t_a} = \mathrm{Re} = \frac{l^*c_0}{\nu} \sim 10^5\mathcal{O}(l^*) >> 1 \tag{S28}$$

It is evident from Eq. S28 that viscosity is too slow compared to wave motion, so the fluid essentially behaves inviscid in the acoustic field.

Next, the time-averaged radiation torque feeds into the slower hydrodynamics physics, resulting in a purely tangential velocity field equal to $u_\theta = \frac{a^2 \omega_{\text{spin}}}{r}$. It's easy to verify that the flow is irrotational, which limits the velocity disturbance to a local effect decaying like $\frac{1}{r}$ away from the cylinder. Knowing that the hydrodynamic pressure field is uniform across the fluid, the wave equation including the hydrodynamic mean flow can be written as [3]

$$\frac{\partial^2 p}{\partial t^2} - {c_0}^2 \nabla^2 p + 2(\boldsymbol{u}_0 \cdot \nabla)\frac{\partial p}{\partial t} + (\boldsymbol{u}_0 \cdot \nabla)^2 p + ((\boldsymbol{u}_0 \cdot \nabla)\boldsymbol{u}_0) \cdot \nabla p = 0 \quad \text{(S29)}$$

The order of magnitude of the first two terms in the LHS of Eq. S29 is $\omega^2 p$, while $2(\boldsymbol{u}_0 \cdot \nabla)\frac{\partial p}{\partial t} \sim 2\epsilon_h \omega^2 p$ and $(\boldsymbol{u}_0 \cdot \nabla)^2 p \sim \epsilon_h^2 \omega^2 p$ are one and two orders of magnitude smaller compared to the original wave equation provided in Eq. 2.6 if $\epsilon_h = \frac{|\boldsymbol{u}_0|}{c_0} = \frac{a\omega_{\text{spin}}}{c_0} << 1$, which is true for all typical values. Moreover, the last term in the LHS of Eq. S29, which arises due to nonuniformity of the flow, has an order of magnitude equal to $\epsilon_h^2 \omega^2 \delta p$ where $\delta = \frac{\lambda}{L_m}$ is the ratio of acoustic wavelength to length scale of mean-flow variation. As the hydrodynamic velocity decays with a rate of $\frac{1}{r}$, a typical value for L_m would be $L_m = a$, which satisfies $\delta << 1$ even in the large-wavelength regime. Accordingly, the acoustics and hydrodynamic physics can be considered as decoupled and Stokes flow formulation can be used to acquire the terminal rotational velocity of the cylinder, as is done in Eq. 3.6.

References

[1] Henrik Bruus. Acoustofluidics 1: Governing equations in microfluidics. *Lab on a Chip*, 11(22):3742–3751, 2011.

[2] Lin Chen and Yuhiro Iwamoto. *Advanced applications of supercritical fluids in energy systems.* IGI Global, 2017.

[3] Allan D Pierce. Wave equation for sound in fluids with unsteady inhomogeneous flow. *The Journal of the Acoustical Society of America*, 87(6):2292–2299, 1990.
